# Loss of TDP‐43 function accelerates tauopathy and death of vulnerable neurons by promoting caspase 3‐mediated endoproteolytic cleavage of tau

**DOI:** 10.1002/alz.087558

**Published:** 2025-01-03

**Authors:** Meghraj Singh Baghel, Grace D Burns, Margarita Tsapatsis, Aswathy P Mallika, Shaelyn R Marx, Tong Li, Philip C Wong

**Affiliations:** ^1^ Johns Hopkins University School of Medicine, Baltimore, MD USA; ^2^ National Institute on Aging, National Institutes of Health, Bethesda, MD USA

## Abstract

**Background:**

TDP‐43 proteinopathy, initially discovered in amyotrophic lateral sclerosis (ALS) and frontotemporal dementia (FTD), coexists with tauopathy in a variety of neurodegenerative disorders, including Alzheimer’s Disease (AD). While such co‐pathology is strongly associated with worsened neurodegeneration and steeper cognitive decline, how these two pathologies influence each other to exacerbate neuron loss remains elusive. That loss of TDP‐43 splicing repression occurring in presymptomatic ALS‐FTD suggests that loss of TDP‐43 function could facilitate the pathological conversion of tau to accelerate tauopathy and neuron loss.

**Method:**

To elucidate how loss of TDP‐43 function influence tauopathy, we used two complementary approaches to express a four‐repeat domain fragment of human tau *(hTauRD)* to seed tauopathy in *CaMKII‐CreER;Tardbp^f^* ^/^ *^f^* mice: 1) inducible transgenesis; and 2) intraparenchymal delivery of an adeno‐associated viral vector unilaterally in the hippocampus.

**Results:**

Here, we found that depletion of TDP‐43 is sufficient to activate caspase 3‐dependent cleavage of tau and cell death of vulnerable neurons, such as those in CA3/2 of the hippocampus, in mice lacking TDP‐43 in their forebrains (*CaMKII‐CreER;Tardbp^f^* ^/^ *^f^* mice). To establish this mechanistic insight that the level of activated caspase 3‐dependent cleavage of tau may determine the vulnerability of neurons in AD cases harboring the co‐pathology of TDP‐43, we elevated levels of hTauRD in *CaMKII‐CreER;Tardbp^f^* ^/^ *^f^* mice to stimulate the activation of caspase 3. As compared to age‐matched controls, we show an additive impact of TDP‐43 loss‐of‐function and hTauRD to promote further increase of activated caspase 3 to elevate the cleavage of endogenous tau, thereby accelerating death of cells to also include neurons of the dentate gyrus and/or CA1.

**Conclusion:**

Taken together with previous results documenting that activated caspase 3‐dependent cleavage of tau occurs in cases of AD and their mouse models, our findings strongly support the view that TDP‐43 loss‐of‐function accelerates tauopathy in vulnerable neurons by promoting caspase 3‐mediated endoproteolytic cleavage of tau, thereby exacerbating neuron loss in neurodegenerative disorders harboring co‐pathologies of tau and TDP‐43, and disclose for these human diseases a potential therapeutic target to attenuate neuron loss.

